# Linking genotype to phenotype in multi-omics data of small sample

**DOI:** 10.1186/s12864-021-07867-w

**Published:** 2021-07-13

**Authors:** Xinpeng Guo, Yafei Song, Shuhui Liu, Meihong Gao, Yang Qi, Xuequn Shang

**Affiliations:** 1 School of Computer Science, Northwestern Polytechnical University, Xi’an, 710072 People’s Republic of China; 2 School of Air and Missile Defense, Air Force Engineering University, Xi’an, 710051 People’s Republic of China

**Keywords:** Multi-omics, Small sample, SNP, Gene, Phenotype

## Abstract

**Background:**

Genome-wide association studies (GWAS) that link genotype to phenotype represent an effective means to associate an individual genetic background with a disease or trait. However, single-omics data only provide limited information on biological mechanisms, and it is necessary to improve the accuracy for predicting the biological association between genotype and phenotype by integrating multi-omics data. Typically, gene expression data are integrated to analyze the effect of single nucleotide polymorphisms (SNPs) on phenotype. Such multi-omics data integration mainly follows two approaches: multi-staged analysis and meta-dimensional analysis, which respectively ignore intra-omics and inter-omics associations. Moreover, both approaches require omics data from a single sample set, and the large feature set of SNPs necessitates a large sample size for model establishment, but it is difficult to obtain multi-omics data from a single, large sample set.

**Results:**

To address this problem, we propose a method of genotype-phenotype association based on multi-omics data from small samples. The workflow of this method includes clustering genes using a protein-protein interaction network and gene expression data, screening gene clusters with group lasso, obtaining SNP clusters corresponding to the selected gene clusters through expression quantitative trait locus data, integrating SNP clusters and corresponding gene clusters and phenotypes into three-layer network blocks, analyzing and predicting based on each block, and obtaining the final prediction by taking the average.

**Conclusions:**

We compare this method to others using two datasets and find that our method shows better results in both cases. Our method can effectively solve the prediction problem in multi-omics data of small sample, and provide valuable resources for further studies on the fusion of more omics data.

**Supplementary Information:**

The online version contains supplementary material available at 10.1186/s12864-021-07867-w.

## Background

An important goal of current genetics is to establish global functional associations between genotype and phenotype, the so-called genotype-phenotype map [1]. The study of genotype-phenotype association sheds new light on the process of genetic variation [2]. Genome-wide association studies (GWAS) that link genotype to phenotype represent an effective way to associate individual genetic backgrounds with specific diseases or traits. The strategy is to locate all the differential sites in a genome and correlate them to a phenotype. Over the past decade, many GWAS have been performed to identify genetic variants associated with complex human diseases or traits. These findings highlight novel associations between variants and traits, provide insight into racial variations in complex traits, and lead to multiple clinical applications [3, 4]. However, most variants explain only a small fraction of the causal genetic factors. According to the principle of GWAS, although thousands of single nucleotide polymorphisms (SNPs) for complex diseases and traits have been identified, single-omics analysis can only provide limited information on biological mechanisms, and the functions and mechanisms of SNP loci remain largely unclear.

Due to limitations at the single-omics level, it is necessary to integrate multi-omics data to more accurately predict the biological associations between genotype and phenotype [5]. Such data allow us to study interactions across omics, and provide opportunities to further examine genotype-phenotype associations and uncover the underlying mechanisms [6]. Gene expression data are typically incorporated to analyze the impact of SNPs on phenotype [7–9]. The two main approaches of multi-omics data integration are multi-staged analysis and meta-dimensional analysis [10, 11].

When using a multi-omics biological network to explore genotype-phenotype association, it is generally believed that the difference in phenotypic traits is primarily attributable to the cumulative effect of omics. For example, SNPs lead to changes in gene expression, which in turn affect protein expression and ultimately cause diseases [12]. Such layer-by-layer integration analysis is usually called multi-staged analysis. The idea is to establish the association between two layers by linear regression, partial least squares (PLS) [13, 14], canonical correlation analysis, and correlation coefficients, and to predict diseases through the associations across layers. Multi-staged analysis bridges the gap between genotype and phenotype by gene expression traits. The most commonly used such approaches are three-layer methods such as the network-driven association mapping (NETAM) algorithm for regression models based on biological networks [15], in which a three-level association network is constructed, linear regression is used to establish the association between SNPs and genes, and logistic regression is used to link genes with phenotypes (with values 0 or 1, representing the absence or presence, respectively, of a disease). Disease prediction by analyzing the effect of SNPs on gene expression is more accurate than direct prediction by SNPs. This confirms that the three-layer network is more likely to reflect real biological relationships. Similar models include PrediXcan [16] and backward three-way association mapping (BTAM) [17]. However, when a regression model is established on a three-layer network, intra-omics correlations are not considered, which results in low model accuracy.

Meta-dimensional analysis has the three approaches of concatenation-based, transformation-based, and single-omics model-based integration. In concatenation-based integration, the features of omics data are integrated through machine learning to form a more comprehensive input matrix, through which a prediction model is established [18]. In transformation-based integration, multi-omics data are transformed to an intermediate form, which is used for integration and development of a predictive model [19]. In single-omics model-based integration, predictive models are established using individual omics data, and their predictions are integrated to produce the final output [20]. A common drawback of meta-dimensional analysis is that the omics data have identical weights, meaning that they are used to predict phenotypes from different perspectives without examining their biological correlations. Although meta-dimensional analysis can improve the accuracy of phenotype prediction, the biological significance of multi-omics data integration remains unclear.

Furthermore, genotype-phenotype association by multi-omics requires omics data from a single sample set, and because of the large feature set of SNPs, both multi-omics analysis methods require larger sample sizes for model construction. Acquisition of clinical data can be hampered by patient privacy protection and specific data requirements of various institutions. Therefore, public clinical data do not meet the needs of multi-omics data integration methods in terms of sample size and number of omics. We propose a method of genotype-phenotype association for multi-omics data based on a small sample size and large feature set. This method offers the following innovations. a) It solves the problem of ineffective regression due to a large feature set and small sample size in three-layer networks. b) It considers intra-omics associations to improve prediction accuracy. c) It analyzes the biological pathway associations across omics layers to clarify the biological significance. d) It accounts for tissue specificity, because each tissue of a multicellular organism has distinguishing characteristics due to the tissue-specific expression of genes that confer unique morphological structures and physiological functions on various tissues [21, 22]. e) In the study of the SNP-gene-phenotype pathway, our method is the most time-saving over state-of-the-art methods.

## Results

### Data sources and preprocessing

Two datasets derived from the Gene Expression Omnibus (GEO) database were used to verify the effectiveness of our method [23]. GSE33356 represents the data of lung adenocarcinoma [24]. Affymetrix SNP 6.0 and Affymetrix U133 Plus 2.0 microarrays were used to analyze the specimens of lung tumors and normal tissues from 84 nonsmoking women with adenocarcinoma. GSE114269 represents the data comparing medullary breast carcinoma (MBC) with non-medullary basal-like breast carcinoma (non-MBC BLC) among 48 patients [25]. These two datasets were selected to show whether our method can be useful in genotype and phenotype classification in various cases of small sample sizes and large feature sets.

The PPI network data were derived from PICKLE (Protein InteraCtion KnowLedgebasE) [26], which are metadata that integrate human PPI from various open sources through gene ontology information. The eQTL data were obtained from GTEx Analysis V7 (dbGaP Accession phs000424.v7.p2) [27]. For accurate data prediction, eQTL data were selected based on tissue specificity. For example, lung eQTL data was selected for the first dataset, and breast tissue eQTL data for the second.

The data were preprocessed for consistent nomenclature of SNPs and genes in data of various types. Data with more than 10% missing values were removed from SNPs, data with less than 10% missing values were imputed with the most frequent value or the mean value, and only SNP data with minor allele frequency (MAF) greater than 0.1 were used.

### Prediction analysis

There is currently no typical method of genotype–phenotype association for data with a small sample size and a large number of features. We compared the following methods to verify the feasibility of our approach. In comparison experiments, the method developed in this paper was named GSPLS (Group lasso and SPLS model). The detailed implementation process of this method can be viewed in the section “Methods” and the Supplementary Information.

1) The method GGLM (Group lasso and Generalized Linear Model) differs from GSPLS in that multiple regression rather than SPLS is used to associate SNP layers with gene layers, which allows us to verify the effect of gene-gene association while considering intra-omics association.

2) The method NETAM [15] involves multi-staged analysis, in which gene clustering are absent. Each SNP group was defined by the SNPs within the transcribed region of a gene, and a large three-layer network is established based on all the data and subjected to direct multiple regression with lasso. This method does not cluster SNPs and genes, and can be used to verify the effect of inter-omics association without gene associating and clustering. NETAM consists of two parts: Sparse Regression and Stability Selection. There are two user-defined parameters T and *π*_*tbr*_ in this method (*π*_*tbr*_: threshold for stability selection, T: total number of random samples). These two parameters serve the Stability Selection part. However, due to our small sample size, Stability Selection cannot be carried out, so we test NETAM without the benefits of stability selection, where lasso and L1-regularized logistic regression are employed with 5-fold cross-validation.

3) The method mixOmics [28] involves multi-omics integration, in which SNP and gene data are used to independently establish prediction models, and predictions are integrated to generate the final output. This method ignores SNP-gene association and allows us to verify the effect of omitting inter-omics association, but it fails to analyze the inter-omics pathway relationship.

**Fig. 1:**
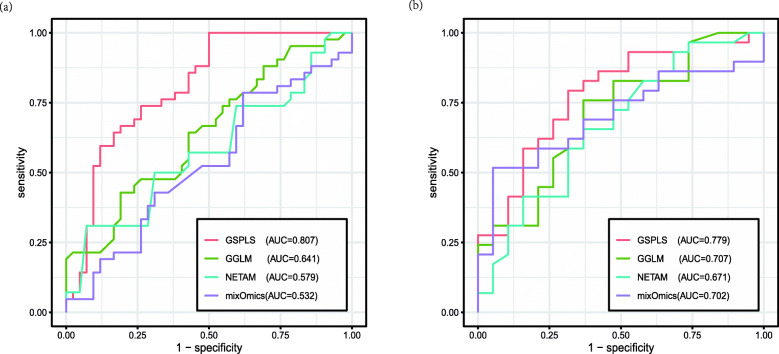
ROC curves to compare the performance of GSPLS with association mapping methods such as GGLM, NETAM, and mixOmics. The left one is the result of analysis on GSE33356, and the right one is the result of analysis on GSE114269. The legend of each figure contains the AUC value of each method on the data. The AUC value of our method is the highest, indicating that our method is superior to other methods

Our method was compared to the above three approaches on GSE33356 and GSE114269 datasets. Because it was a dichotomous classification, receiver operating characteristic curves were used for comparison and explanation. Figure 1 shows the area under curve for the four methods.

As shown in Fig. 1, our method achieved better results on both datasets. The performance of GSPLS was significantly improved over that of GGLM, indicating that intra-omics associations should be considered in the analysis to better fit real biological systems. Both GSPLS and GGLM with gene network clustering showed superiority over other methods, indicating that clustering and screening gene data followed by taking the average are suitable for processing data from small samples. The NETAM method showed the worst performance, possibly because it is not suitable for small samples, but it provides guidelines for inter-omics pathway analysis. It has been documented that when analyzing SNP-gene-phenotype associations, this algorithm is suitable when the sample size reaches 500. In the case of a small sample, it is ineffective in regression. The result of mixOmics was similar to that of GGLM, especially on the second dataset. In our test, it sometimes even outperformed GGLM, but this method ignores inter-omics associations.

In this experiment, we found that the results of the last three methods were unstable with small sample sizes. Overall, GSPLS was superior to GGLM, mixOmics, and NETAM with small sample sizes.

Through the selection of gene clustering and corresponding SNP clusters, we not only grouped the sample features, but also screened the grouped features through Group Lasso, so that the feature quantity contained in each block was sharply reduced, which not only reduced the sample size required by our algorithm, but also reduced the computation time. We tested the four methods GSPLS, GGLM, NETAM and mixOmics on GSE33356 data with 10 repeated experiments respectively on a PC with Intel Core i7-10510U processors, 16 Gb RAM, and NVIDIA GeForce MX330 video card. The computation time of GSPLS was 66.213 ± 3.905 s, GGLM was 138.527 ± 12.149 s, NETAM was 712.141 ± 89.491 s and mixOmics was 47.065 ± 4.135 s. Although the GSPLS time is longer than the mixOmics time, the mixOmics cannot reflect the pathway correlation. In the study of the SNP-gene-phenotype pathway, our method is the most time-saving.

### Sample sizes analysis

In the study of three-way association analysis among genotypes, gene traits and phenotypes, large number of samples are often needed. For example, NETAM [15] showed significantly better performance (larger area under the curve) for *N* > 200, but GSPLS only needs dozens of samples.

**Fig. 2:**
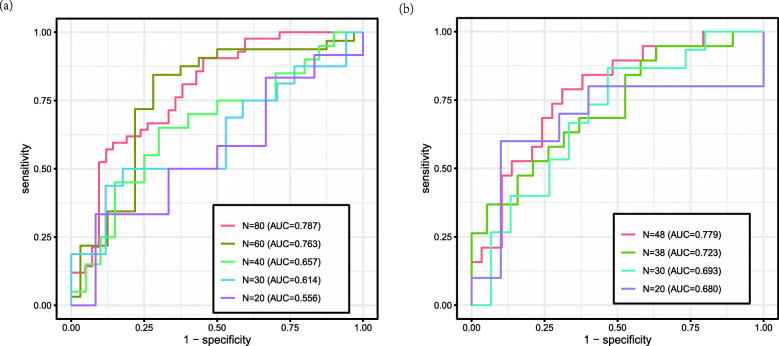
ROC curves to compare the performance of GSPLS with different sample sizes. The left one is the result of analysis on GSE33356, and the right one is the result of analysis on GSE114269. The legend of each figure contains the sample sizes and the AUC value of each sample size on the data. With the decrease of the sample size, the corresponding AUC value also showed a decreasing trend

To validate the effect of sample size, we randomly select samples *N* = 80, 60, 40, 30, 20, from the data set GSE33356, and *N* = 48,40,30,20, from the data set GSE114269 (Because this data set contains only 48 sample sizes). The positive and negative sample sizes are guaranteed to be equal during extraction. When sample size is less than 20, Group Lasso could not achieve effective regression for these two data sets. Therefore, the minimum sample size we choose is 20. In Fig. 2, we show receiver operating characteristic (ROC) curves that show true positive and false positive rates of the results produced by GSPLS with different sample sizes. The results show that the general trend of AUC value decreases with the decrease of sample size. Although this method is suitable for small sample data, unlike NETAM, which requires a sample size of more than 200, it also requires a sample size of more than 20.

### Pathway analysis

We analyzed the generated pathways to further demonstrate the effectiveness of our method. The weight of the edge between each gene and relevant SNP in the cluster was obtained by SPLS, and the weight of the edge connecting disease and the relevant gene was obtained by group lasso. The two layers of weights were multiplied to yield the weight value of a pathway (if no edge was present, then the edge weight was set to zero), which we name Pathway Score (PS) to reflect its importance.

**Fig. 3:**
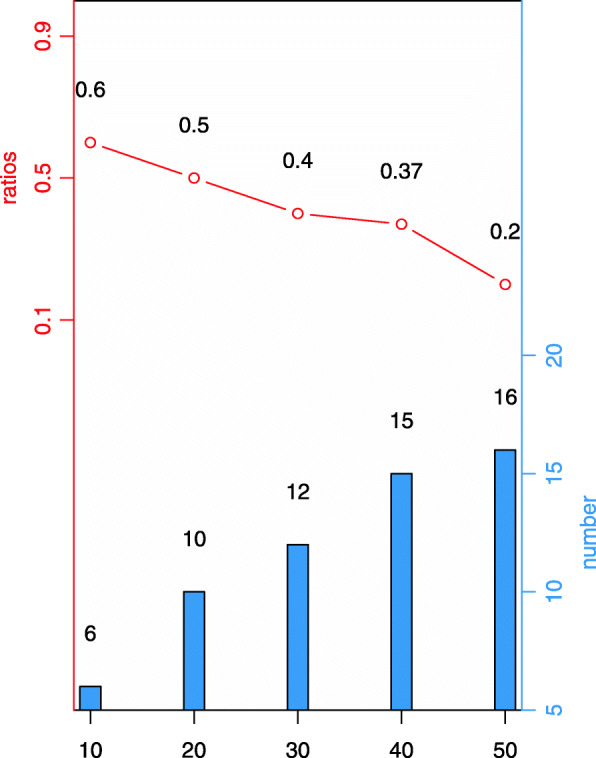
Double ordinate statistical analysis for the generated pathways. The abscissa represents the number of the pathways ranked by PS. The vertical axis of the red line on the left represents the proportion of SNPs or genes matched in the PhenoScanner database, while the vertical axis of the blue line on the right represents the number of SNPs or genes matched in the PhenoScanner database. For example, SNPs or genes in 6 of the top 10 pathways were present in PhenoScanner, the proportion is 0.6

Using GSE33356 as an example, we ranked the generated associations by PS, and aligned the top 50 pathways with the lung cancer-associated SNPs and genes in the PhenoScanner database [29], which contains 137 genotype-phenotype association datasets, as well as association catalogs such as NHGRI-EBI GWAS, NHLBI GRASP, and dbGaP. Alignment against PhenoScanner reflects the distribution of SNPs or genes in the pathways obtained by this method (Fig. 3).

As shown in Fig. 3 and Table 1, We found that the percentage of SNPs or genes present in PhenoScanner decreased as the total number of PS ranks increased, mainly because when numerically solving pathway relationships, SNPs or genes with the same trend are randomly interchanged during data sparsification. With the decrease of PS, the total number of SNPs and genes with the same trend increased, the possibility of interchanging SNPs or genes matching with the database increased, and thus the percentage of such SNPs or genes decreased.

**Table 1:** PS values of the top 20 pathways

	SNP	Gene	PS	Presence in PhenoScanner
1	rs12419692	BAG5	0.0971	Yes
2	rs1794429	F2	0.0887	Yes
3	rs7615840	THBD	0.0854	No
4	rs363082	THBD	0.0810	No
5	rs4150581	BAG5	0.0784	Yes
6	rs7615840	PROS1	0.0747	Yes
7	rs5751141	XRCC6	0.0574	Yes
8	rs5751141	ZHX1	0.0544	No
9	rs5758464	HGFAC	0.0500	Yes
10	rs1794429	SLC30A2	0.0496	No
11	rs10503418	WWC1	0.0485	Yes
12	rs4346818	METTL27	0.0389	No
13	rs12467784	ERBB4	0.0332	Yes
14	rs11250130	MTMR6	0.0329	Yes
15	rs2470615	HIF3A	0.0303	No
16	rs4824079	HNRNPDL	0.0298	No
17	rs6502780	PSME8	0.0253	Yes
18	rs4346818	NCAPH2	0.0247	No
19	rs738202	VEGFD	0.0245	No
20	rs1331057	FGG	0.0237	No

## Discussion

We addressed genotype–phenotype association based on multi-omics data for small samples. In the case of data with numerous features, there are two commonly used approaches: dimension reduction and feature selection. However, the former may lead to the inability to analyze features before dimension reduction. Therefore, sparsification and existing associations were used to screen and reduce the number of features. The greatest contribution of this paper is the ability to group genes into clusters according to their network characteristics while significantly reducing the number of features in each cluster. The clustering and effective screening of gene networks drastically reduces the number of features of the three-layer network, making it suitable for application to data with small sample sizes.

Data is a key in multi-omics studies. However, the following two conditions generally occur to the multi-omics data. One is that with the increase of the types of omics data, the multi-omics data is not easy to obtain and the data sample size is not large. In this paper, such problems can be solved in the case of small samples, the advantage is that it can reflect the association relationship of each omics, it makes the biological mechanism clearer, but the accuracy is not high. The other is that when the sample size is large, the number of omics types is often small, such as GWAS data. Although GWAS analysis has a certain accuracy, it is generally analyzed by statistical methods without understanding the internal biological mechanism. How to achieve the analysis effect of multi-omics data, when we use the data with sufficient sample size and insufficient omics types. This will be the direction of our next research.

Our method has obvious advantages, but the data rely on the PPI network and eQTL association. Therefore, only associations in an SNP/CNV-gene-phenotype three-layer network can be processed, rather than any three omics, such as SNP, methylation, and phenotype.

When clustering with SPICi, although we can select the ranges of the three clustering hyperparameters according to certain criteria, unknown factors still influence the selection of range limits, and this method is highly sensitive to the choice of hyperparameters. We also attempted to replace SPICi clustering with fuzzy clustering, which showed results similar to sampling multiple gene clusters with replacement and was expected to achieve better clustering. However, fuzzy clustering requires specification of parameters, including cluster number. Inappropriate initial parameters may affect clustering accuracy, and the real-time performance of the algorithm can be compromised by a large sample dataset and feature set. These problems must be addressed in future studies.

## Conclusion

In summary, in this study we used SPICi and Group Lasso methods to perform gene cluster and screen, obtained the SNP corresponding to gene in each cluster through eQTL data, established the association relationship between SNP and gene by SPLS, and formed block together with phenotype. Finally, these blocks are integrated by taking the average. Compare with state-of-the-art methods, our method achieved better results on GSE33356 and GSE114269 datasets, and it consumes the least time. To validate the effect of sample size, we randomly select samples from these two datasets, we found that our method was effective for the sample size greater than 20. And we ranked the generated associations by PS, SNPs or genes in 6 of the top 10 pathways were present in PhenoScanner, as were those in 10 of the top 20 pathways. Collectively, our method can effectively solve the prediction problem in multi-omics data of small sample, and provide valuable resources for further studies on the fusion of more omics data.

## Methods

### Algorithms

**Fig. 4:**
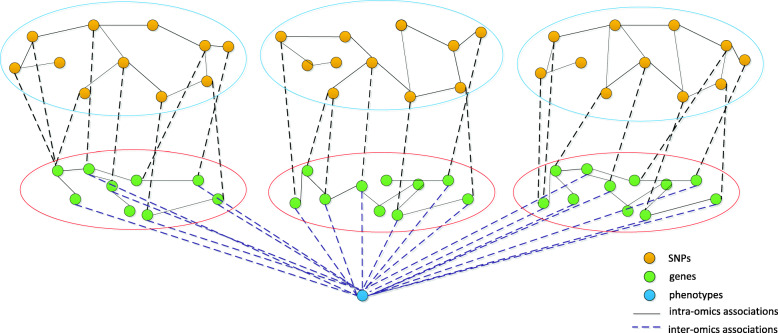
Model of genotype-phenotype association based on data integration algorithm. Red oval represent gene cluster, and blue oval represent SNP cluster. Gene association network is clustered to generate gene clusters. SNP clusters corresponding to the selected gene clusters can be identified by eQTL data. Each gene cluster, correlated SNP cluster, and phenotype are combined in a three-layer network, achieving the purpose of rapid dimensionality reduction

Multi-staged analysis ignores intra-omics correlations and requires a large sample size, whereas meta-dimensional analysis ignores inter-omics associations. To address this issue, we propose an approach to genotype-phenotype association for small samples based on multi-omics data. The model is illustrated in Fig. 4.

**Fig. 5:**
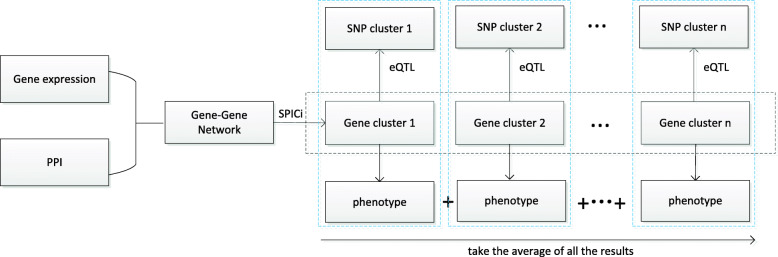
Flowchart of genotype-phenotype association based on data integration algorithm. The steps of this method in this figure can be described as follows: 1) PPI network and gene expression data are used to generate a weighted undirected gene association network, which is clustered by SPICi to generate gene clusters; 2) Gene clusters are screened by group lasso; 3) SNP clusters corresponding to the selected gene clusters are obtained from eQTL data; 4) SNP clusters, correlated gene clusters, and phenotype are combined in a three-layer network block, which is used for prediction and analysis through three-layer associations; 5) Results from various blocks are integrated by taking the average to produce the final output

Diseases are not caused by the effects of one or more genes [30–32], but by gene interactions. A protein–protein interaction (PPI) network can reflect gene–gene associations, but this network is unweighted. Gene expression data can be used to calculate the Pearson correlation coefficient between genes, which serves as the weight of an edge, and this can be combined with a PPI network to produce a weighted gene network. Closely related genes are more likely to interact and impact on a phenotype, so we performed speed and performance in clustering (SPICi) on a gene association network to obtain multiple gene clusters (see section “Clustering method selection and hyperparameter setting”) [33]. Due to the large number of genes, and thus the many obtained gene clusters, group lasso regression can be performed on gene clusters and phenotypes. Gene clusters with nonzero coefficients are most likely to have impacts on a disease, and this regression serves the purpose of gene cluster screening. SNP clusters corresponding to the selected gene clusters can be identified by expression quantitative trait loci (eQTL) that control the expression level of quantitative trait genes. It is important to note that the same SNP may occur in multiple SNP clusters. In this way, correlated SNP clusters, gene clusters, and phenotypes are combined in a three-layer network, which is called a block. The SNP and gene layers can be linked by sparse partial least squares (SPLS) [14], and gene layers and phenotypes by logistic regression. The prediction and analysis of blocks are based on three-layer associations (see section “Three-layer network construction”). The predictions of blocks are integrated by taking the average. The flowchart is shown in Fig. 5. Additional details are provided in the Supplementary Information.

### Clustering method selection and hyperparameter setting

Clustering algorithms play an important role in the analysis of biological networks and can reveal their functional modules. Most clustering algorithms perform well on biological networks of moderate size, but they become impractical on larger networks due to low speed or high network complexity. According to the analysis of Jiang et al. for each clustering methods including Markov cluster (MCL) [34], molecular complex detection (MCODE) [35], Cfinder [36], the memory-constrained unweighted pair group method using arithmetic averages (MCUPGMA) [37], and dense module enumeration (DME) [38], SPICi [33] was selected to cluster the generated gene network. SPICi uses a heuristic approach to greedily build clusters, which is orders of magnitude faster, and was the only method to successfully cluster all test networks in a short period of time [33]. Its three hyperparameters are minimum cluster size, minimum support threshold, and minimum cluster density, which jointly affect the number of clusters and the number of elements in each cluster. To facilitate subsequent analyses, the final numbers of clusters and elements should fall in appropriate ranges. If there are too many elements in a cluster, during group lasso calculation the goal-oriented error caused by a given penalty parameter is greater for a group with more elements. If there are too few elements in a cluster, the effect of gene association on disease cannot be effectively analyzed. Generally, gene data were clustered into 100–200 clusters, and each cluster containing between 5 and 100 genes. We further analyzed the settings of the three hyperparameters as they have different characteristics.

The minimum cluster size is used to determine the inclusion/exclusion of a cluster by comparing the number of genes involved in it. If the number of elements in a cluster is greater than the minimum cluster size, then the cluster is included. If the minimum cluster size is too small, then it fails to capture the associations between genes, and the opposite may result in the inappropriate deletion of clusters. According to tests on different datasets, the minimum cluster size was set to the interval of [13, 14].

In an undirected graph *G* = (*V*, *E*), for any vertex *u* and connecting vertex set *S* ⊂ *V*, *support* is defined as

$$ support\left(u,S\right)={\sum}_{v\in S}{w}_{u,v}, $$

i.e., the sum of weights of all edges connected to vertex *u*, where *w*_*u*, *v*_ is the weight of any edge (*u*, *v*) ∈ *E*. If the *support* of a vertex is less than the minimum support threshold, then the vertex is discarded. The weight of an edge is represented by the Pearson correlation coefficient of two vertex vectors; a coefficient can be positive or negative, and coefficients can cancel each other during the calculation of *support*. Therefore, the absolute value of the Pearson correlation coefficient was used, so *w*_*u*, *v*_ ∈ (0, 1]. Generally, the absolute value of Pearson correlation coefficient is less than 0.2, which indicates very weak correlation or no linear correlation, so we only take the edges whose absolute value of Pearson correlation coefficient is greater than 0.2 to form the gene-gene network. If a vertex is only linked to a gene with weak association, then additional noise may be introduced, so it is required that if a vertex is only linked to genes with weak association, then two or more such edges should be present, so the lower limit of the minimum support threshold was set to 0.4. In tests with various datasets, when the minimum support threshold value was greater than 0.7, the total number of genes became insufficient to reflect the impact of gene association on disease. Therefore, the minimum support threshold was set to the interval of [0.4, 0.7].

Cluster *density* is defined as the sum of edge weights divided by the total number of possible edges, and it is used to reflect the density of subgraphs. It is calculated as

$$ density(S)=\frac{\sum_{u,v\in S}{w}_{u,v}}{\mid S\mid \ast \left(|S|-1\right)/2}. $$

As shown in the formula, too small a *density* increases the number of elements in each cluster and decreases the total number of clusters. When the *density* of a cluster is less than the minimum cluster density, SPICi clusters it into two or more clusters. Therefore, the minimum cluster density directly affects the total number of clusters and has the greatest impact on clustering among the three hyperparameters. Based on comparative experiments, the minimum cluster density was set to [0.1, 0.6], and the parameter was tested in increments of 0.1 during the experiment. Through testing, we determined the hyperparametric settings of the two data sets in our paper, as shown in Table 2.

**Table 2:** The hyperparametric settings on the two data sets

	minimum cluster density	minimum support threshold	minimum cluster size
GSE33356	0.1	0.4	5
GSE114269	0.2	0.5	5

### Three-layer network construction

**Fig. 6:**
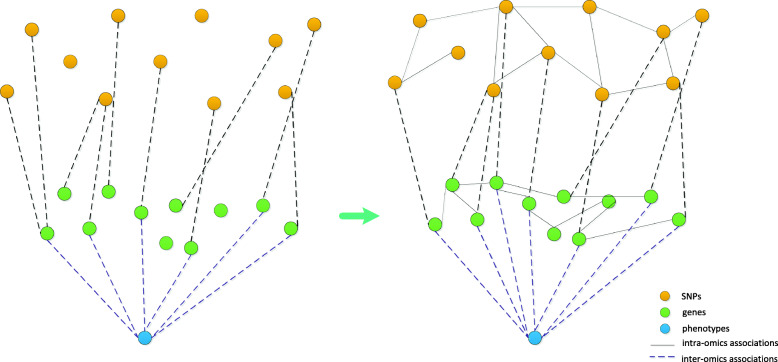
Model of genotype-phenotype association based on biological network. The left one is the schematic diagram without considering the intra-omics associations, and the right one is the schematic diagram used in our method. It contains both intra-omics associations and inter-omics associations, which can better reflect the biological reality. Without considering the intra-omics associations, the model cannot truly reflect the biological reality, and without considering the inter-omics associations, it cannot well reflect the whole biological system

Gene clusters closely related to a disease can be selected by group lasso [39, 40]. The causal link from these gene clusters to a disease is established by related differential sites in the genome. The corresponding SNP clusters can be obtained by eQTL data. Each SNP cluster as well as the correlated gene cluster and phenotype can be combined in a three-layer network block. After dividing into blocks, the number of SNPs and gene features in the three-layer structure decreases drastically, as does the sample size required for effective regression, which facilitates regression of numerous features with small sample size. When analyzing the three-layer structure of each block, although interlayer regression alone can predict some pathway relationships, it does not consider intra-omics associations, and it deviates from biological reality. However, to only consider intra-omics associations and ignore inter-omics pathway correlations fails to reflect the whole biological system and represents only the local situation (Fig. 6). Therefore, when we look at the associations between SNPs and genes, the associations of individual elements between layers (left part of Fig. 6) were expanded to intra- and inter-layer associations (right part of Fig. 6). That is, the many-to-one associations were broadened to many-to-many associations. Accordingly, the solution was altered from multiple regression to SPLS, which incorporates a penalty function in PLS [14]. The principle of PLS is as follows. With *q* dependent variables {*y*_1_, …, *y*_*q*_} and *p* independent variables {*x*_1_, …, *x*_*p*_} [41], the statistical relationships between dependent and independent variables can be studied by observing *n* sample points, which constitute the data matrices *X*(*n* × *p*) and *Y*(*n* × *q*) of independent and dependent variables, respectively. PLS regression extracts principal components *t*_1_ and *u*_1_ from *X* and *Y*, respectively. For regression analysis, *t*_1_ and *u*_1_ should carry as much information as possible of the data they represent. After extraction of the first set of principal components *t*_1_ and *u*_1_, PLS performs regression of *X* on *t*_1_ as well as *Y* on *u*_1_. If the regression equations are sufficiently accurate, then the algorithm is terminated. Otherwise, a second round of component extraction is performed using the residual information after removing the variance of *X* and *Y* explained by *t*_1_ and *u*_1_, respectively. This process is repeated until satisfactory accuracy is achieved. SPLS combines the advantages of principal component analysis, canonical correlation, and linear regression, and effectively solves the problems of ineffective regression and multicollinearity among features due to fewer samples than features. In order to assess the impact and usefulness of the single steps, GSE33356 is used as an example data to analyze the intermediate results of each step in the Supplementary Information.

## Supplementary Information

**Additional file 1.**

## Data Availability

The method is available at https://github.com/2017100647/GSPLS. Publicly available datasets were analyzed in this study. This data can be found here: https://www.ncbi.nlm.nih.gov/geo/query/acc.cgi?acc=GSE33356; https://www.ncbi.nlm.nih.gov/geo/query/acc.cgi?acc=GSE114269; http://www.pickle.gr/; https://gtexportal.org/home/datasets.

## Abbreviations

GWAS: Genome-wide association studies
SNPs: single nucleotide polymorphisms
BTAM: backward three-way association mapping
PPI: protein–protein interaction
SPICi: speed and performance in clustering
eQTL: expression quantitative trait loci
SPLS: sparse partial least squares
GEO: Gene Expression Omnibus
MBC: medullary breast carcinoma
non-MBC BLC: non-medullary basal-like breast carcinoma
PICKLE: Protein InteraCtion KnowLedgebasE
MAF: minor allele frequency
ROC: receiver operating characteristic
PS: Pathway Score
