# Supplementary material for: Linking genotype to phenotype in multi-omics data of small sample
Source: BMC Genomics. 2021 Jul 13;22:537. doi: 10.1186/s12864-021-07867-w (PMC8278664; doi:10.1186/s12864-021-07867-w)
Supplement: Supplementary file 1 — Additional file 1. [file 12864_2021_7867_MOESM1_ESM.docx]

**Supplementary Information: Linking Genotype to Phenotype in Multi-omics Data of Small Sample**

Xinpeng Guo

## Source code

Source code for analysis is available at: https://github.com/2017100647/GSPLS

## Experimental process analysis

In order to assess the impact and usefulness of the single steps, GSE33356 is used as an example data to analyze the intermediate results of each step.

**Data**

GSE33356 represents the data of lung adenocarcinoma. Affymetrix SNP 6.0 and Affymetrix U133 Plus 2.0 microarrays were used to analyze the specimens of lung tumors and normal tissues from 84 nonsmoking women with adenocarcinoma (42 tumor, 42 adjacent-normal).

The PPI network data (UniProt IDs: 16384, Interactions: 214446) were derived from PICKLE ( <http://www.pickle.gr/Downloads#HUMAN-3-2> ).

The eQTL data were obtained from GTEx Analysis V7 (dbGaP Accession phs000424.v7.p2). For accurate data prediction, eQTL data were selected based on tissue specificity. For example, lung eQTLs were selected for GSE33356 data.

**Preprocessing**

Gene data preprocessing. Rows represent genes and columns represent samples in gene raw data. Gene name is Affymetric probe name. firstly, we convert gene name to Gene Symbol, and if there is the same gene name, the average value of gene expression is taken. There are 22971 rows of data, which means the data contains 22971 genes.

SNP data preprocessing. Rows represent SNPs and columns represent samples in SNP original data in SNP raw data. We require that the missing values should not exceed 10%. If it is greater than 10%, we will delete this row and less than 10% should be filled with the most frequent data. Then SNPs with minor allele frequency<0.01 were filtered. There are 496415 rows of data, which means the data contains 496415 SNPs.

The eQTL data will be named in the form of RS and Gene in the form of Symbol. There are 648939 eQTL data.

The association relationship of PPI network was mapped to gene association relationship. The absolute value of Pearson's correlation coefficient (w) is used for the weight of edges (w>0.2). There are 156223 gene association data. The connection relationship of edges comes from PPI network, PPI network was mapped to gene association relationship. The weight of edges is the Pearson correlation coefficient of two nodes, Pearson correlation coefficient is solved by the Cor () function of R. We only take the edges whose absolute value of Pearson correlation coefficient is greater than 0.2 to form the gene-gene network (Generally, the absolute value of Pearson correlation coefficient is less than 0.2, which indicates very weak correlation or no linear correlation).

After naming unification, the intersection of SNP data and eQTL data can be obtained by SNP name, and the intersection of Gene data, PPI data and eQTL data can be obtained by Gene name. For example, SNP names will be matched to remove the SNP data that are not in eQTL data.

At this moment SNP data has 194463 rows, gene data has 12220 rows, and there are 371622 eQTL data and 66408 gene association data.

**Gene clustering**

Gene-gene networks are clustered by the SPICi clustering algorithm ( <https://compbio.cs.princeton.edu/spici/> ). In the manuscript, we explain the three hyperparameter ranges of SPICi method. Through the test, it is found that when minimum cluster density = 0.1, minimum support threshold = 0.4, and minimum cluster size = 5, the clustering results obtained are helpful for subsequent analysis. Through this step, gene data were clustered into 190 clusters, each cluster containing between 5 and 80 genes.

**Group lasso**

Due to the large number of gene clusters, group lasso regression can be performed on gene clusters and phenotypes. Gene clusters with nonzero coefficients are most likely to have impacts on a disease, and this regression serves the purpose of gene cluster screening. We do this work with Package ‘grpreg’ of R language. It is very easy to use this package, because it has no hyperparameters. For details, refer to the group-spls.R file in my code file. In the last, we get 15 clusters.

**SNP clusters**

SNP clusters corresponding to the selected gene clusters can be identified by eQTLs that control the expression level of quantitative trait genes. Therefore, using the eQTL data, we obtained the corresponding 15 SNP clusters from 15 gene clusters, each SNP cluster containing between 200 and 600 SNPs.

eQTL data is the data reflecting the association relationship between SNP and gene. When we have the gene clusters after screening, we can generate the corresponding SNP clusters only using eQTL data. The association relationship between SNPs and genes may be many-to-many in eQTL data, so one SNP may appear in multiple SNP clusters. For example, SNP1 is associated with both Gene1 and Gene2, and Gene1 may be in the gene cluster Class1, while Gene2 may be in the gene cluster Class2, and the corresponding SNP cluster of Class1 will contain SNP1, and the corresponding SNP cluster of Class2 will also have SNP1, in this way, one SNP can affect multiple genes or gene clusters.

**SPLS method and Output**

Correlated SNP clusters, gene clusters, and phenotypes are combined in a three-layer network, which is called a block. SNP and gene were calculated in each block, the relationship between SNP and gene was calculated by SPLS method, the relationship between gene and phenotype was calculated by logical regression method. We averaged the results of 15 blocks to produce the final output.

**Alternative Methods**

1. **GSPLS**

According to the principle of GWAS, single-omics analysis can only provide limited information on biological mechanisms, and the functions and mechanisms of SNP loci remain largely unclear. Extending the two-way association (i.e. genotypes and gene traits) analysis, three-way association analysis among genotypes, gene traits and phenotypes also has been proposed. three-way association analysis provide opportunities to further examine genotype-phenotype associations and uncover the underlying mechanisms, so we present a novel method, GSPLS, to detect ‘path associations’ from SNPs to phenotypes through gene expression traits. Therefore, the methods for comparison are mainly three correlation analysis methods of SNP-gene-phenotype.

1. **GGLM**

The only difference between GGLM and GSPLS is in solving the association relationship between SNP and gene, and GGLM method adopts multiple regression t to establish the relationship model between SNP and gene.

1. **NETAM**

NETAM was derived from this literature (Lee S, Kong S, Xing EP. A network-driven approach for genome-wide association mapping. Bioinformatics. Jun 15 2016;32(12):i164-i173.). NETAM consists of two parts: Sparse Regression and Stability Selection. There are two user-defined parameters T and $\pi_{tbr}$in this method ($\pi_{tbr}$: threshold for stability selection, T: total number of random samples). These two parameters serve the Stability Selection part. However, due to our small sample size, Stability Selection cannot be carried out, so we test NETAM without the benefits of stability selection, where lasso and L1-regularized logistic regression are employed with 5-fold cross-validation.

1. **mixOmics**

mixOmics method was derived from this literature (Rohart F, Gautier B, Singh A, Lê Cao KA (2017) mixOmics: An R package for 'omics feature selection and multiple data integration. PLOS Computational Biology 13(11): e1005752.).
